# Evaluation of the Antioxidative and Hepatoprotective Activity of *Ulva lactuca* in a CCl_4_-Induced Liver Injury Model

**DOI:** 10.3390/foods15152695

**Published:** 2026-07-30

**Authors:** Noohela Khan, Waqas Ahmed, Muhammad Asif Ali, Imtiaz Rabbani

**Affiliations:** 1 Department of Food Science and Human Nutrition, University of Veterinary and Animal Sciences, Lahore 38040, Pakistan; nohail.khan3@gmail.com (N.K.); asif.ali@uvas.edu.pk (M.A.A.); 2 Department of Physiology, University of Veterinary and Animal Sciences, Lahore 38040, Pakistan; imtiaz.rabbani@uvas.edu.pk

**Keywords:** antioxidant, functional foods, hepatoprotective, macroalgae, nutraceuticals, phytochemicals, seaweed, *Ulva lactuca*

## Abstract

**Background**: *Ulva lactuca* is abundant in bioactive components that provide antioxidative, anti-inflammatory and hepatoprotective properties against oxidative stress. **Objectives**: This study aimed to explore the nutritional composition, antioxidative potential and hepatoprotective efficacy of *U. lactuca* in a CCl_4_-induced liver injury model. **Methods**: Proximate analysis, phytochemical profiling (phenolic and flavonoid content, GC-MS and LC-MS) and antioxidant assays (DPPH and FRAP) were performed, followed by an in vivo efficacy trial. Hepatotoxicity was induced using CCl_4_, and *U. lactuca* methanolic extract (1000 mg/kg body weight) was administered for four weeks. **Results**: The proximate analysis demonstrated 10.5% ash, 10.83% protein and 39.43% fibre. GC-MS and LC-MS identified hexadecanoic acid methyl ester, gallic acid, quercetin, quercitrin, 4-hydroxybenzoic acid and syringic acid. TPC was higher in aqueous (2015.1 mgGAE/100 mL) and TFC in ethanolic (750.5 µgQE/mL) extract. Aqueous extract (170.1 ± 0.1 µmol/g) displayed superior reducing power while ethanolic extract (77.7%) exhibited the highest radical scavenging activity. *U. lactuca* promoted weight recovery, improved lipid profiles, decreased hepatic and oxidative stress biomarkers (*p*-value < 0.05), and restored liver histology. **Conclusions**: *U. lactuca* showed promising effects against sub-chronic liver injury, primarily due to its rich nutritional profile. Thus, its incorporation into the Pakistani diet may reduce disease and economic burden while supporting functional food and nutraceutical industries.

## 1. Introduction

Oxidative stress is the major contributing factor in chronic liver disease. Reactive oxygen species generated during oxidative stress lead to the disruption of hepatic structure, necrosis of hepatic cells, prognosis of regenerative nodules and replacement of normal hepatic parenchyma with fibrotic tissue [1]. Moreover, oxidative stress plays a critical role in the development of liver disorders, including hepatitis, fibrosis and cirrhosis. Recent epidemiological studies indicate that more than 1.5 billion people are affected by chronic liver diseases annually [2], accounting for 1.8% of global disease burden [3]. Thus, this highlights the need for effective hepatoprotective strategies to mitigate disease development and progression.

Conventional hepatoprotective drugs, while effective, often pose challenges related to their side effects, cost and limited accessibility. These limitations have increased interest in natural products, particularly from marine sources, due to their therapeutic potential [4]. Recently, marine-derived compounds have gained immense attention from the pharmaceutical, functional food and nutraceutical industries primarily due to their rich biologically active profile. This has caused a global increase in the demand for marine-based functional foods.

Marine macroalgae have long been essential to the diets and traditional medicine of coastal populations worldwide, particularly in Asian countries [5]. Seaweeds are rich sources of phytochemicals such as polyphenols (caffeic acid and syringic acid), flavonoids (quercetin and kaempferol), sulphated polysaccharides (ulvan and fucoidans), pigments like chlorophyll, lutein, β-carotene and fucoxanthin, sterols, minerals such as iodine, magnesium, iron and calcium, both soluble and insoluble dietary fibres, and polyunsaturated fatty acids that are associated with antioxidative, anti-inflammatory, hypolipidemic and hepatoprotective properties [6,7]. Polyphenols and flavonoids are the prime secondary metabolites that provide these protective effects, especially hepatoprotective properties, by reducing oxidative stress through various mechanisms including radical scavenging, the downregulation of inflammatory pathways, the inhibition of lipid peroxidation and the enhancement of natural defence systems [8]. These are generally characterised as Chlorophyta (green), Phaeophyta (brown) and Rhodophyta (red) based on the biochemical composition and pigmentation [9,10]. Among green seaweed, *Ulva lactuca* has gained notable attention because of its high nutritional value [9,10].

The global market for marine-derived pharmaceuticals, functional foods and nutraceuticals has shown an exponential growth in the recent years and is projected to reach USD 7.1 billion by the end of 2026 and USD 9.64 billion by 2030, with a CAGR (compound annual growth rate) of 7.5% and 8%, respectively [11]. The global seaweed market is expected to exceed 30 billion in 2030 with a CAGR of approximately 10.7%. Despite the global surge, the current contribution of Pakistan still remains limited, with total marine seaweed exports of USD 2.1 million in 2022 [12]. However, as per the recent report on the growth rate of Pakistan’s seaweed market, the domestic seaweed market is still in its early stages with a current growth rate of 1.5%, and it is expected to reach around 2.49% by 2029 [13].

Various environmental factors such as geography, temperature, salinity, nutrient availability and water depth strongly influence the nutritional profile and therapeutic potential of seaweed [14]. Moreover, due to their nutrient-rich profile, seaweeds are the main focus of the functional food and nutraceutical markets. Despite global interest in seaweed-derived nutraceutical and functional foods, especially in Japan, South Korea and China, comprehensive nutritional, phytochemical and therapeutic studies on Pakistani seaweed species have not been conducted [15]. Thus, this has caused a significant knowledge gap that necessitates localised investigation.

Therefore, this study was designed to provide a comprehensive evaluation of *Ulva lactuca* from Pakistani coastal areas. The study integrates a nutritional (proximate composition) analysis, compositional characterisation (including total phenolic and flavonoid content, and GC-MS and LC-MS profiling), an antioxidant assessment (DPPH and FRAP) and in vivo validation to investigate the hepatoprotective potential of *Ulva lactuca*. The study aims to address the existing research gap and highlight the novelty of utilising *U. lactuca* as a functional food ingredient or nutraceutical candidate for the management of oxidative-stress-related liver disorders, while contributing to the sustainable utilisation of the marine resources of Pakistan.

## 2. Materials and Methods

The research was structured in two phases: preliminary analyses and in vivo efficacy trial.

### 2.1. Procurement and Sample Preparation

#### 2.1.1. Raw Material and Sample Collection

*Ulva lactuca* was identified and collected from the coastal areas of Karachi, Pakistan, after the confirmation of a botanist from Government College University, Lahore.

#### 2.1.2. Sample Preparation

Collected samples underwent thorough 3–5 rinsing cycles using distilled water to eliminate salt impurities and debris. After washing, the samples were air-dried under controlled laboratory conditions (25 ± 2 °C) until a constant weight was achieved to ensure uniform moisture content prior to grinding. The samples were then finely pulverised into powder and passed through a 60-mesh sieve to obtain a particle size of ≤250 µm. Then, the powdered samples were stored in polyethene bags and kept in dark conditions at room temperature to protect from humidity and light. This ensured their preservation for further utilisation [16].

#### 2.1.3. Chemicals and Reagents

All the chemicals and reagents used in the current study were of analytical grade and were purchased from Thermo Scientific Chemicals (Waltham, MA, USA) and Sigma-Aldrich (St. Louis, MO, USA).

### 2.2. Phase 1: Preliminary Analyses

The preliminary analyses included proximate analysis, phytochemical analyses and antioxidant assays.

#### 2.2.1. Proximate Analysis

Proximate analysis of *U. lactuca* was performed according to the methods mentioned in the Association of Analytical Chemists (AOAC) International protocols. Moreover, each test was conducted thrice to ensure accuracy. The parameters examined included moisture content (method 930.04), total ash content (method 930.05), crude protein (using the Kjeldahl method; method 978.04), ether extraction of crude fat (method 930.09), crude fibre (method 930.10) and, lastly, nitrogen-free extract (NFE) [17]. As per the previous study, 6.25 was applied as the conversion factor to calculate the crude protein [18]. Supplementary Table S1 represents the formulas used for the calculation of each component.

#### 2.2.2. Extract Preparation

Methanol, ethanol and aqueous extracts of *Ulva lactuca* were prepared using the maceration extraction technique with slight modifications [19]. Dried seaweed samples (10 g) were soaked in 100 mL of the respective solvent and placed in Eyela MMS-220H orbital shaker for 24 h at 30 °C with a shaking speed of 143 rpm. After that, the seaweed samples were filtered using a Whatman filter (paper grade No. 1) and concentrated using Heidolph Hei-VAP Core rotary evaporator (Heidolph Instruments GmbH & Co. KG, Schwabach, Germany) equipped with G1 diagonal glassware condenser at 40 °C with a rotation speed of 40 rpm and under reduced pressure of 10 mmHg. They were stored at 4 °C for further analysis. Moreover, the yield was calculated to determine the extraction efficiency of *Ulva lactuca* methanol, ethanol and aqueous extracts and expressed as a percentage [20]:

$$\mathrm{Extract}\;\mathrm{Yield}\;(\%)\;=\;(\frac{Weight\;of\;solvent\;free\;extract}{Weight\;of\;sample})\;\times 100$$

#### 2.2.3. Phytochemical Analyses

##### Gas Chromatography–Mass Spectrometry (GC-MS)

For the compositional analysis of *Ulva lactuca*, gas chromatography–mass spectrometry using an Agilent 5977 Single Quadrupole GC-MS system (Agilent Technologies, Santa Clara, CA, USA) was performed. Data were acquired using MassHunter GC-MS acquisition software version 10.0.0368. Furthermore, the acquired spectra were assessed against the NIST-20, a National Institute of Standards and Technology mass spectral library, for compound identification.

##### Liquid Chromatography–Mass Spectrometry (LC-MS)

The phytochemical profiling of *Ulva lactuca* was performed using the Agilent 6410A triple quadrupole system (Agilent Technologies, Santa Clara, CA, USA). Moreover, the data were acquired using Agilent MassHunter acquisition software version 10.0 (Build 142).

##### Phytochemical Profile: Total Phenolic and Flavonoid Content

The Folin–Ciocalteu method was utilised to estimate the total phenolic content in methanolic, ethanolic and aqueous extracts of *U. lactuca*. Methodology involved the formation of a blue complex upon the reaction of phenolic compounds with Folin–Ciocalteu reagent. The absorbance was assessed at 765 nm, utilising a Cary 60 UV-Vis spectrophotometer (Agilent Technologies, Santa Clara, CA, USA), and the results were expressed as mg of GAE/100 mL of extract [19].

The total flavonoid content in methanolic, ethanolic and aqueous extracts of *U. lactuca* was measured using the aluminium chloride colourimetry. The procedure included the formation of a flavonoid–aluminium complex measured at 440 nm using a Cary 60 UV-Vis spectrophotometer (Agilent Technologies, Santa Clara, CA, USA) and reported as µg QE/mL of extract [19].

#### 2.2.4. Antioxidant Assays: DPPH (2,2-Diphenyl-1-Picrylhydrazyl) and FRAP (Ferric Reducing Antioxidant Power) Assay

The antioxidant capability of the ethanolic, methanolic and aqueous extracts of *Ulva lactuca* was assessed using the DPPH radical scavenging activity assay, following established protocols with slight modifications [19]. First, a DPPH stock solution was prepared by dissolving 13.01 mg of DPPH in 100 mL of methanol. This solution was then aliquoted into conical tubes (10 mL each) and stored at −20 °C for future use. For each analysis, a working solution was prepared by thawing 10 mL of the stock DPPH solution (330 μM) and diluting it with 20 mL of methanol. The absorbance of this working solution as a control was adjusted to 1.10 ± 0.02 units at 515 nm using a Cary 60 UV-Vis spectrophotometer (Agilent Technologies, Santa Clara, CA, USA), with methanol serving as the blank. To evaluate the free radical scavenging activity, 100 μL of the sample extract was mixed with 1400 μL of the DPPH working solution. The mixture was then incubated at 37 °C for 30 min. After incubation, absorbance was computed at 515 nm using a Cary 60 UV-Vis spectrophotometer (Agilent Technologies, Santa Clara, CA, USA) against blank. The antioxidant activity was quantified in terms of Trolox equivalents (mg/L), using a standard calibration curve prepared in methanol, with a correlation coefficient (R^2^) exceeding 0.98. Lastly, the results were reported as percentage (%) using the below-mentioned formula:

$$DPPH\;radical\;scavenging\;activity\;(\%)=\frac{\left(Absorbance\;of\;the\;control-Absorbance\;of\;the\;sample\right)}{Absorbance\;of\;the\;control}\times 100\;$$

An FRAP, i.e., ferric reducing antioxidant power assay, was utilised to evaluate the reducing power capacity of *Ulva lactuca* methanolic, ethanolic and aqueous extracts [21]. The method relies on the reduction of ferric ions (Fe^3+^) to ferrous ions (Fe^2+^) by antioxidants in the sample. For this purpose, various reagent solutions were freshly prepared. The acetate buffer (300 mM, pH 3.6) was prepared by mixing 10 mL of 0.3 M sodium acetate solution (2.46 g sodium acetate in 100 mL distilled water) with 90 mL of 0.3 M acetic acid solution (1.72 mL acetic acid in 100 mL distilled water). A 40 mM hydrochloric acid (HCl) solution was prepared by mixing 0.05 mL of concentrated HCl with 3.75 mL of distilled water and adjusting the final volume to 15 mL. For the preparation of the 10 mM TPTZ (2,4,6-tripyridyl-s-triazine) solution, 15.62 mg of TPTZ was dissolved in 5 mL of the HCl solution. The ferric chloride solution (20 mM) was prepared by dissolving 27.03 mg of ferric chloride in 5 mL of distilled water. The FRAP working reagent was prepared freshly before each analysis by mixing 25 mL of acetate buffer with 2.5 mL of TPTZ solution and 2.5 mL of FeCl_3_ solution. Prior to use, both the FRAP reagent and distilled water were pre-warmed to 37 °C in a water bath. To perform the assay, 100 μL of the extract was added to 700 μL of distilled water and 700 μL of the warmed FRAP reagent. The mixture was incubated at 37 °C for 4 min. The FRAP assay led to the formulation of a blue-coloured ferrous–tripyridyltriazine complex. After incubation, absorbance was measured at 593 nm using a Cary 60 UV-Vis spectrophotometer (Agilent Technologies, Santa Clara, CA, USA), with distilled water serving as the blank. The antioxidant activity was initially expressed as millimoles of Fe^2+^ equivalents (mMFeSO_4_), based on a standard curve constructed using ferrous sulfate (FeSO_4_) in water, with a correlation coefficient (R^2^) greater than 0.98. However, to ensure compatibility with the international standards, the results were recalculated as µmol/g, i.e., µmol Fe (*II*) per gram of dry extract.

Phase 2: In Vivo Efficacy Trial

Twenty-four male healthy Wistar albino rats (150–200 g) were obtained from the University of Veterinary and Animal Sciences, Lahore, and acclimatised for one week under the controlled conditions (20–25 °C, 12 h cycle of light/dark) with standard pellet diet and ad libitum water intake. Following ethical approval from the institution’s Ethical Review Committee (ERC No. DR/122), rats were randomly allocated into four experimental groups (n = 6 per group). Furthermore, there were no exclusions. Hepatotoxicity was induced by intraperitoneal injections of carbon tetrachloride (CCl_4_), a well-established model that reliably triggers oxidative stress and liver damage by generating free radicals, thereby mimicking key features of human liver disease [22]. Silymarin served as a standard drug, and treatment group received *U. lactuca* methanolic extract for four weeks (Table 1).

Assessment

The antioxidative and hepatoprotective potential of *Ulva lactuca* was evaluated using a comprehensive set of biochemical and physiological parameters including body weight, lipid profile parameters, liver function markers, oxidative stress biomarkers, inflammatory markers, and atherogenic indices (Supplementary Table S2) [23,24]. Blood samples were collected via cardiac puncture under anaesthesia. Lastly, histopathology of liver tissues was performed [25].

### 2.3. Statistical Analysis

IBM SPSS version 28 for the statistical analyses and GraphPad Prism version 10.4.2 for graph generation were utilised. One-way ANOVA followed by Duncan’s multiple range test as a post hoc test was applied at the threshold of ≤0.05. Results are presented in graphical and tabular formats as percentages using the mean and standard deviation.

## 3. Results

### 3.1. Phase 1: Preliminary Analyses

#### 3.1.1. Proximate Analysis

Table 2 represents the proximate analysis of *U. lactuca* including the total moisture content (8.97 ± 0.06%), total ash content (10.50 ± 0.44%), total protein (10.83 ± 0.05%), crude fat (2.75 ± 0.25%), crude fibre (39.43 ± 3.95%) and NFE, i.e., nitrogen-free extract (27.52 ± 3.49%).

#### 3.1.2. Extraction Yield

**Figure 1:**
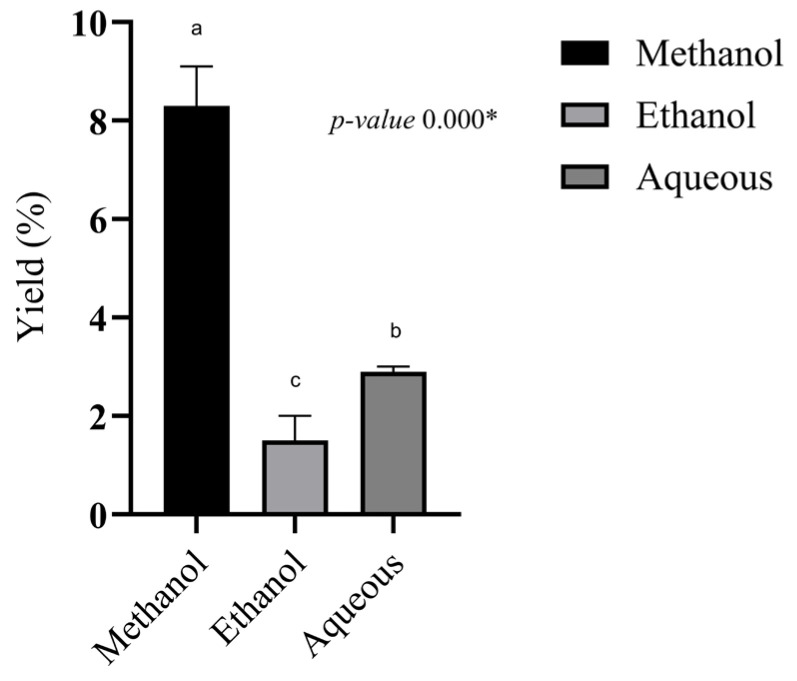
Extract yield of *Ulva lactuca* (all values are presented as percentages (%) and expressed as mean ± standard deviation (n = 3)). Multiple superscripts letters above the bars indicate statistically significant differences. * Statistically significant *p*-value ≤ 0.05.

Figure 1 displays a yield comparison of *Ulva lactuca* methanolic, ethanolic (1.5 ± 0.5%) and aqueous extracts (2.9 ± 0.1%), with methanolic extract exhibiting the highest value at 8.3 ± 0.8% with a *p*-value of 0.000.

#### 3.1.3. Phytochemical Analysis

##### Gas Chromatography–Mass Spectrometry (GC-MS)

The GC-MS analysis of the methanolic and ethanolic extracts of *Ulva lactuca* identified several bioactive compounds at different retention times and percentage peaks (Table 3, Supplementary Figure S1). The methanolic and ethanolic extracts revealed the presence of hexadecanoic acid, methyl ester as a prominent component, exhibiting antioxidative, anti-inflammatory, antimicrobial, antifibrotic and hepatoprotective properties.

##### Liquid Chromatography–Mass Spectrometry (LC-MS)

The LC-MS analysis of methanolic (Table 4 and Supplementary Figure S2), ethanolic (Table 4 and Supplementary Figure S3) and aqueous (Table 4 and Supplementary Figure S4) extracts revealed the presence of distinct phenolic compounds and flavonoid compounds. The methanolic extract confirmed the presence of six compounds belonging to the class of phenolic acids, including gallic acid, caffeic acid sulphate, caffeic acid, 4-hydroxybenzoic acid, syringic acid and vanillin. Additionally, the extract revealed the presence of three compounds belonging to the class of flavonoids, including kaempferol, quercetin and quercitrin. Moreover, the ethanolic extract disclosed the presence of a total of seven compounds, mainly phenolic acids, including 4-hydroxybenzoic acid, vanillic acid, caffeic acid, gallic acid and syringic acid, and flavonoids such as quercetin and quercitrin. Lastly, the aqueous extract was also found to be abundant in flavonoids (kaempferol, quercetin and quercitrin) and phenolic acids (syringic acid, vanillic acid, 4-hydroxybenzoic acid, caffeic acid and gallic acid).

#### 3.1.4. Phytochemical Profile

Table 5 displays a significant difference among various extracts, with aqueous (2015.1 ± 0.6 mg GAE/100 mL) and ethanolic extract (2014.6 ± 0.9 mg GAE/100 mL) exhibiting greater phenolic content (*p*-value: 0.000), and ethanolic extract (750.5 ± 0.7 µg QE/mL) exhibiting greater flavonoid content (*p*-value: 0.000).

#### 3.1.5. Antioxidant Assays

Table 5 represents a significant difference among various extracts, with the antioxidant profile of the ethanolic extract exhibiting the highest radical scavenging activity (77.7 ± 0.02%), while the aqueous extract had superior reducing power (170.1 ± 0.1 µmol/g) (*p*-value: 0.000).

### 3.2. Phase 2: In Vivo Efficacy Trial

#### 3.2.1. Body Weight

Table 6 represents the impact of the different interventions on the weight of the experimental rats throughout the study. The normal control group showed an increasing trend in body weight (*p*-value: 0.000). In contrast, the disease control group exhibited a stable body weight (*p*-value: 0.201). The interventions with the standard drug and *Ulva lactuca* interventions significantly affected the rats’ body weight with varying degrees of effectiveness (*p*-value: ≤ 0.05).

#### 3.2.2. Serum Lipid Profile

Table 7 presents the positive impact of different treatments on the lipid profile of the experimental rats. The results showed that the macroalgae significantly (*p*-value: <0.05) improved the components of their lipid profile, including total cholesterol (133.2 ± 5.7 mg/dL), TGs (99.2 ± 7.7 mg/dL), HDL (22.4 ± 6.5 mg/dL), LDL (90.9 ± 7.9 mg/dL), VLDL (19.8 ± 1.5 mg/dL) and cholesterol-to-HDL ratio (6.4 ± 1.9) in comparison to the disease control group.

#### 3.2.3. Atherogenic Indexes

The results presented in Table 8 illustrate the impact of various interventions on atherogenic indexes across different groups. The results suggested that *Ulva lactuca* improved the atherogenic index parameters (*p*-value: 0.000), including the atherogenic index of plasma (0.66 ± 0.14), Castelli’s Risk Index I (6.38 ± 1.92), Castelli’s Risk Index II (4.42 ± 1.59) and the atherogenic coefficient (5.38 ± 1.92), and contributed positively to the rats’ cardiovascular health compared to the disease control group.

#### 3.2.4. Hepatic Function Panel

Table 9 shows the impact of the algal treatment on hepatic function parameters in different rat groups. The results indicated that *U. lactuca* as an intervention significantly reduced the levels of total bilirubin (0.96 ± 0.11 mg/dL), ALT (62.57 ± 6.04 U/L), AST (117.87 ± 5.97 U/L), ALP (333.17 ± 4.02 U/L) and GGT (3.76 ± 0.32 U/L), while improving the total protein (6.88 ± 0.38 g/dL), albumin (3.09 ± 0.29 g/dL) and globulin (3.79 ± 0.27 g/dL) levels. All the parameters showed statistically significant differences with a *p*-value for each component of 0.000 compared to the disease control group, indicating the effectiveness of *U. lactuca*.

#### 3.2.5. Impact of Various Interventions on Inflammatory and Oxidative Stress Biomarkers

**Figure 2:**
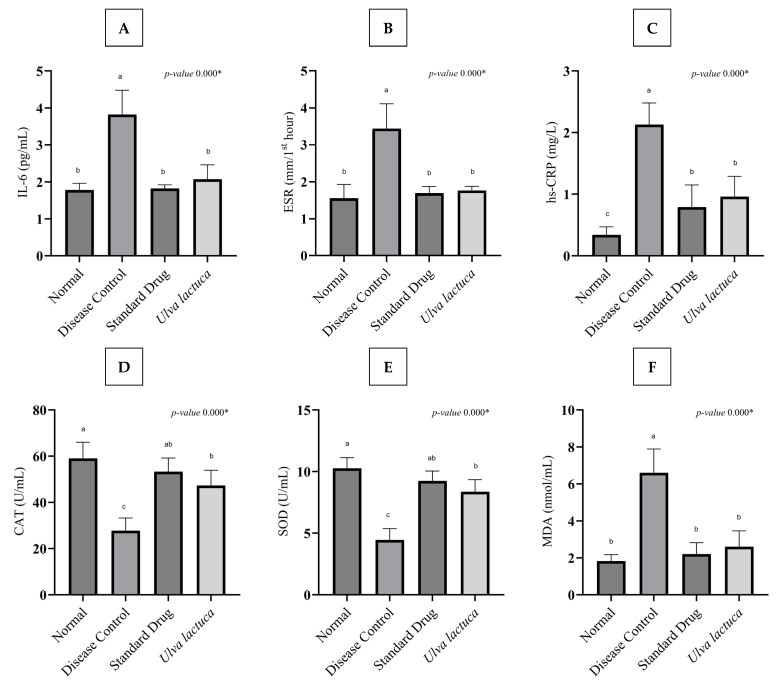
Impact of various interventions on inflammatory and oxidative stress biomarkers. Effect on IL-6 (interleukin-6) (**A**); Effect on ESR (erythrocyte sedimentation rate) (**B**); Effect on hs-CRP (high-sensitivity C-reactive protein) (**C**); Effect on catalase (CAT) (**D**); Effect on superoxide dismutase (SOD) (**E**); Effect on malondialdehyde (MDA) (**F**). All the data are represented as mean ± standard deviation. Various superscript letters indicate statistically significant differences between the groups. * Statistically significant *p*-value ≤ 0.05.

Figure 2 summarises the impact of various interventions on inflammatory markers, i.e., IL-6 (interleukin-6), hs-CRP (high-sensitivity C-reactive protein) and ESR (erythrocyte sedimentation rate), and oxidative stress biomarkers, including CAT (catalase), SOD (superoxide dismutase) and MDA (malondialdehyde). CAT and SOD are antioxidant enzymes, and MDA is the compound produced as a result of lipid peroxidation. The results demonstrated that the disease control group exhibited the highest levels of IL-6 (3.82 ± 0.66 pg/mL), ESR (3.44 ± 0.67 mm/1st hour) and hs-CRP (2.13 ± 0.35 mg/L), suggesting elevated inflammation. In contrast, the algal interventional group demonstrated a significant reduction in IL-6 (2.07 ± 0.39 pg/mL), ESR (1.76 ± 0.12 mm/1st hour) and hs-CRP (0.96 ± 0.33 mg/L), thus revealing the anti-inflammatory potential of *U. lactuca* compared to the disease group (*p-*value: 0.000).

Likewise, the catalase levels were drastically reduced in disease control group II (27.73 ± 5.51 U/mL). In contrast, normal control group I (59.05 ± 7.01 U/mL) showed the highest levels of catalase in serum. Moreover, the standard drug (53.3 ± 5.88 U/mL) and *U. lactuca*-treated (47.28 ± 6.64 U/mL) groups had significantly improved catalase levels. Similar trends were observed in the superoxide dismutase levels measured in serum. The SOD levels peaked in the normal control group at 10.26 ± 0.87 U/mL, and the disease control group (4.45 ± 0.92 U/mL) had the lowest levels. The *U. lactuca* interventional group efficiently reduced the levels to 8.36 ± 0.98 U/mL, disclosing a marked decrease in the oxidative stress. Lastly, the opposite trend was seen as the MDA levels were notably elevated in the disease control group (6.60 ± 1.29 nmol/mL), indicating lipid peroxidation and disease occurrence. However, the algal-treated group showed a marked reduction in MDA levels (2.60 ± 0.86 nmol/mL), nearly reaching normal control group levels (1.82 ± 0.35 nmol/mL). The decrease in malondialdehyde levels exhibited the efficiency of *U. lactuca*.

#### 3.2.6. Histopathology of Liver

**Figure 3:**
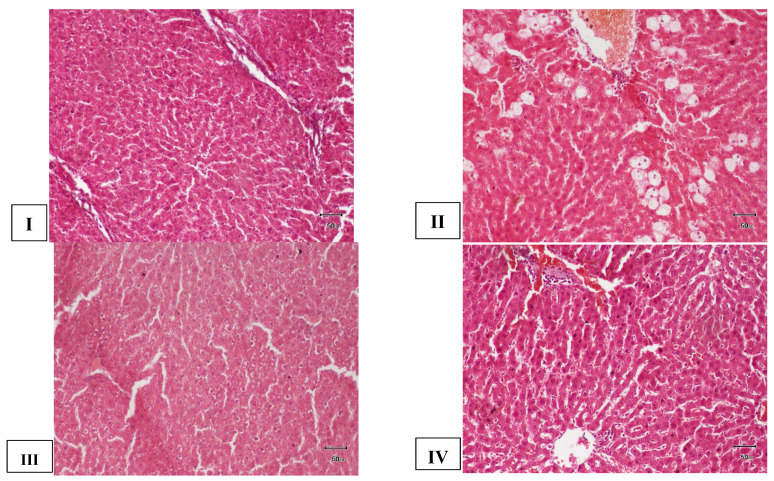
Histopathological examination of liver at 10 × 10 magnification. Normal Control (**I**); Disease Control (**II**); Standard Drug (**III**); *Ulva lactuca* (**IV**). Scale bar: 50 µm.

Histopathology of the livers of the experimental rats was performed and analysed at 10X (Figure 3). Group I (normal control) displayed a normal hepatic structure. Hepatocytes were organised in orderly plates radiating from intact central veins with distinct nuclei and eosinophilic cytoplasm. Group II (disease control) revealed that the hepatocytes in the centrilobular area showed microvesicular and macrovesicular fatty changes. The central veins were congested, and a mild infiltration of mononuclear inflammatory cells was seen in the portal areas as well as in the centrilobular area, showing marked degeneration and hepatic injury.

Group III (standard drug) showed moderate recovery with an intact portal area, but with mild-to-moderate cellular swelling and congestion in the sinusoidal capillaries and in the central vein. Lastly, Group IV (*Ulva lactuca*) exhibited the marked recovery of hepatocyte architecture, closely resembling that of Group I. Hepatocytes maintained consistent shape and size. However, mild degeneration and mild infiltration of mononuclear inflammatory cells were observed in centrilobular and portal areas.

## 4. Discussion

The present study explored the nutrition profile and antioxidative potential of *Ulva lactuca* and evaluated its therapeutic potential in mitigating CCl_4_-induced liver dysfunction. The findings supported the role of antioxidant rich *U. lactuca* as a potential hepatoprotective agent. *Ulva lactuca* is abundant in various bioactive compounds, including flavonoids, and its phenolic compounds play a pivotal role in lowering levels of oxidative stress associated with developing chronic diseases, suggesting macroalgae as a potent antioxidant [47,48].

A proximate analysis provided a thorough overview of the basic nutritional composition of *Ulva lactuca*. A study reported that 100 g of *U. lactuca* contains 15.29 ± 0.03 g moisture, 36.78 ± 1.75 g crude fibre, 20.16 ± 0.16 g crude protein, 1.37 ± 0.05 g crude lipid and approximately 17.17 ± 0.62 g ash [49]. Likewise, another investigation disclosed that it is composed of 6.32 ± 0.25% moisture, 28.91 ± 1.21% ash, 14.36 ± 0.23% crude protein, 0.45 ± 0.01% crude fat and 62.06 ± 0.60% carbohydrates [50]. Comparably, the current investigation revealed that green seaweed contains 8.97% moisture, 10.5% ash, 10.83% crude protein, 2.75% fat, 39.43% fibre and 27.52% NFE. These findings indicate that the proximate composition of *U. lactuca* is comparable to that found in neighbouring countries. However, slight variations can be due to environmental and geographical factors [14]. A limitation in the current study is the use of 6.25 as a conversion factor to calculate the crude protein content, which was applied for consistency with the previous study evaluating the nutritional content of *Ulva lactuca* [18]. The existing literature suggests that the use of the traditional nitrogen-to-protein conversion factor of 6.25 may overestimate the actual protein content due to the presence of non-protein nitrogenous components, such as nucleic acid and chlorophyll in seaweeds. Therefore, a factor of five would have calculated a lower protein content [51]. However, this methodological choice does not impact the interpreted efficacy of the macroalgae in mitigating CCl_4_-induced hepatic injury, as its bioactive potential is primarily attributed to polyphenols.

The primary purpose of the extract yield calculation is to evaluate the extraction efficacy. A study conducted in India disclosed that *U. lactuca* methanolic extract yielded 53.46% [52]. Likewise, in another study, aqueous and ethanolic extracts had almost similar yields of 3.723 mmol/L and 3.552 mmol/L [40]. In the current study, the *Ulva lactuca* methanolic, ethanolic and aqueous extracts revealed percentage yields of 8.3%, 1.5% and 2.9%, respectively. Furthermore, the type of solvent, extraction technique, time and temperature can cause variation in the extraction yield [53]. Additionally, the methanolic extract showed a higher yield percentage, indicating it to be an effective solvent for extraction because of its high polarity and ability to dissolve bioactive components [54]. Therefore, the methanolic extract was selected for further evaluation in the in vivo study.

GC-MS revealed the existence of various polyphenolic and flavonoid compounds in *U. lactuca*. These bioactive metabolites include Hexadecanoic acid [55,56,57], 2,4-Di-tert-butylphenol, Benzeneacetic acid, alpha.,3,4-tris[(trimethylsilyl)oxy]-, methyl ester and Benzo[h]quinoline, 2,4-dimethyl- [26,34,35,36,39]. Furthermore, seaweeds showed the presence of sesquiterpene, Liguloxide and 4(15)-Selinene-11,12-diol, with antimicrobial, anti-inflammatory and antioxidative potential [37,38]. Another polycyclic aromatic compound, Benzo[h]quinoline, 2,4-dimethyl-, with antibacterial, antiviral and anti-inflammatory properties, was reported [39]. Indole alkaloid derivatives like 1H-Indole, 1-methyl-2-phenyl- and 2-Methyl-7-phenylindole with antioxidative and antitumor properties were also detected [31,32,33]. Lastly, another compound, 1-Benzazirene-1-carboxylic acid, 2,2,5a-trimethyl-1a-[3-oxo-1-butenyl] perhydro-, methyl ester, exhibiting antitumor, antioxidant, antimicrobial, antidiabetic and anti-inflammatory potential, was identified [28,29,30]. All the compounds provide hepatoprotection by neutralizing free radicals, modulating inflammatory pathways and restoring the body’s natural antioxidant enzymes. Moreover, recent studies confirmed that these synergistic pathways reduce lipid peroxidation and maintain cell integrity while preserving hepatic architecture [8,58].

The LC-MS analysis of various extracts of *U. lactuca* confirmed that it is abundant in several phenolic components, including gallic acid, syringic acid, caffeic acid, caffeic acid sulphate, 4-hydroxy benzoic acid, vanillin and vanillic acid. These bioactives are potent in providing anti-inflammatory, antioxidative, antimicrobial, antidiabetic, hepatoprotective, neuroprotective, antifungal, anticancerous and cardioprotective effects [40,43]. Additionally, in various studies, it has been disclosed that green seaweed extracts show the occurrence of flavonoids such as kaempferol, quercetin and quercitrin, which possess anti-inflammatory, antiallergic, antioxidative, neuroprotective, cardioprotective and anticancerous properties [40,41,42,44,45,46].

Recently, a study found that the phenolic and flavonoid contents in ethanolic extract were 0.802 mg GAE/g dry weight and 9.157 mg RE/g dry weight, respectively [59]. Another study reported TPC levels of 16.94 mg PGE/g dry weight, TFC levels of 25.7 mg quercetin/g, a total antioxidant activity of 52.18% and a reducing capacity ranging between 1250 and 2500 µg/mL, revealing strong antioxidative potential [60]. Likewise, a recent study disclosed that methanolic and ethyl acetate extracts had antioxidant activities (IC50) of 0.65 ± 0.21 and 0.62 ± 0.01, respectively [43]. Furthermore, the aqueous extract indicated the highest activity with an IC50 of 0.09 ± 0.12. Similarly, the antioxidant capacity (in terms of IC50) of *Ulva lactuca* methanolic extract was found to be more than 1000 µg/mL with a reducing power of 68.69 ± 9.98 [61]. The ferric reducing power of green seaweed was 153.40 ± 1.41 mgGAE/g [62]. Comparably, in the current study, the total phenolic content (TPC) was greater in the aqueous and ethanolic extract, and the total flavonoid content (TFC) was greater in the ethanolic extract. Moreover, the ethanolic extract had the highest radical scavenging activity, while the aqueous extract had superior reducing power.

A safety study disclosed that the algal interventions maintained the weight of the rats [63]. Likewise, another research also revealed that green seaweed caused a weight gain of 14.9 ± 3.4% in hypercholesterolemic rats [64]. Comparatively, the current study exhibited that the algal-treated group showed recovery by regaining their weight following the intervention period. In contrast, the rats in the disease group had a continuous decline in weight throughout the trial from week 1. The weight gain in the final week of the disease group might be attributed to potential fluid accumulation or the adaptive response of the liver [65]. However, as direct indicators such as ascites volume or abdominal girth were not quantified, this observation remains a tentative hypothesis to be explored in more detail in further studies.

A group of researchers showed that *Ulva lactuca* intervention is a promising treatment for the recovery of all hepatic injury biomarkers, improving the lipid profile of subjects [66]. Studies have disclosed that green seaweed showed promising results in improving the lipid profile and lipid ratios of subjects [64,67]. Moreover, various studies have also supported this notion, as *U. lactuca* extract was shown to significantly improve the subjects’ lipid profile with reduced atherogenic indices, and hepatic functionality was shown to have recovered by improving hepatic markers [68,69,70,71]. In comparison, the current study results also align with the findings of these studies by showing improved levels of hepatic markers, demonstrating *U. lactuca*‘s effectiveness against hepatic injury.

Inflammatory markers, for instance, ESR, i.e., the erythrocyte sedimentation rate, CRP, i.e., C-reactive protein, and cytokines, including interleukins and tumour necrotic factor-α, increase during inflammation [72]. A recent study explored the anti-inflammatory potential of *U. lactuca* and revealed that ESR levels dropped after supplementation, thus exhibiting reduced inflammation. The present study showed similar results with a decrease in ESR, IL-6 and C-reactive protein levels [71,73].

A recent study on *U. lactuca* antioxidative properties against nephrotoxicity and hepatotoxicity disclosed a significant improvement in the MDA and SOD levels in liver and kidney homogenates [74]. Similarly, in another study exploring *U. lactuca’s* polysaccharide’s protective effect against oxidative stress revealed its positive impact due to improved SOD and MDA levels [75]. Likewise, another group of researchers also disclosed that *U. lactuca* at various doses reduced malondialdehyde levels and recovered catalase and superoxide dismutase status [76]. The current study also exhibited similar results by significantly managing the SOD, CAT and MDA levels in the *U. lactuca-*treated group.

The liver histopathological analysis of rats intoxicated with D-Galactosamine revealed a reduction in lipid droplets and a moderate level of mitochondrial swelling. In contrast, the disease control group showed prominent lipid accumulation and swelling [77]. Similar therapeutic effects were observed in the current study, revealing a marked improvement in the hepatic structure in comparison to the disease group.

Previous studies on *U. lactuca* are limited to general in vitro analyses, including basic phytochemical analyses, and lack a detailed analysis identifying major bioactive components. Moreover, there are limited data available in detailed compositional and in vivo analyses of Pakistani species. Therefore, the current study bridges the gap and provides a comprehensive nutritional (proximate composition) and compositional characterisation (including total phenolic and flavonoid content and gas and liquid chromatography–mass spectrometry profiling), an antioxidant assessment (DPPH and FRAP) and in vivo validation to investigate the hepatoprotective potential of *Ulva lactuca*.

Lastly, the present study demonstrates that it is rich in various bioactive components, including hexadecanoic acid, methyl ester, gallic acid, quercetin, quercitrin, 4-hydroxybenzoic acid and syringic acid. Moreover, *U. lactuca* exerts a significant hepatoprotective effect against CCl_4_-induced sub-chronic liver injury, as evidenced by the improved lipid profile and decreased hepatic and oxidative stress biomarkers, which were further validated by a histopathological examination of the liver. These findings suggest that *U. lactuca* may serve as a potent nutritional intervention for early-stage hepatic damage. However, further studies utilising long-term chronic liver injury models are needed to explore its efficacy against advanced fibrosis and cirrhosis.

Despite its promising findings, the current investigation has certain limitations. The use of a general nitrogen-to-protein conversion factor of 6.25 for crude protein estimation may not accurately reflect the true protein content of *U. lactuca*, although it was applied for consistency with the previous studies. In addition, the present study employed a sub-chronic liver injury model; therefore, further research using longer-duration chronic liver injury models is required for the better evaluation of *U. lactuca‘s* efficacy against fibrosis and cirrhosis.

## 5. Conclusions

Conclusively, the current investigation supports the antioxidative potential of *Ulva lactuca*. The green seaweed intervention significantly improved the subjects’ lipid profile and decreased hepatic and oxidative stress biomarkers, which were further validated by a histopathological examination of the liver. These findings highlight the promising role of green macroalgae as a potential source for the formulation of nutraceutical products aimed at the management of oxidative-stress-induced liver dysfunction, thereby supporting the growth of the functional food and nutraceutical industries in Pakistan. Although the study demonstrated the notable hepatoprotective effects of *U. lactuca*, extending the duration of the intervention may lead to more noticeable recovery.

## Figures and Tables

**Table 1:** Experimental design for evaluating hepatoprotective effects of *Ulva lactuca* extracts in CCl_4_-induced hepatotoxicity in rats.

Group	Treatment	Hepatotoxicity Induction
I	Standard pellet diet + ad libitum water	None
II	Standard pellet diet + ad libitum water	0.5 mL/kg body weight CCl_4_ (99% purity; Thermo Scientific Chemicals), diluted 1:1 (*v*/*v*) in highly refined pure olive oil (Thermo Scientific Chemicals). Administered via i.p. injection twice weekly (Monday and Thursday) for 4 weeks [22].
III	Standard pellet diet + ad libitum water + silymarin (100 mg/kg p.o. in normal saline)	
IV	Standard pellet diet + ad libitum water + *Ulva* extract (1000 mg/kg p.o.)	

I: Normal control; II: disease control; III: standard drug; IV: *Ulva lactuca*.

**Table 2:** Proximate composition of *Ulva lactuca.*

Proximate Components	*Ulva lactuca* (%)
Moisture	8.97 ± 0.06
Total Ash	10.50 ± 0.44
Crude Protein	10.83 ± 0.05
Crude Fat	2.75 ± 0.25
Crude Fibre	39.43 ± 3.95
Nitrogen-Free Extract (NFE)	27.52 ± 3.49

All values are expressed as percentages (%) on the basis of dry weight (DW) and presented as mean ± standard deviation (n = 3).

**Table 3:** List of identified bioactive compounds from GC-MS profiling of *Ulva lactuca* extracts.

Extract	Peak No.	Retention Time (min)	Compound Name	Molecular Formula	Molecular Weight (g/mol)	Area (%)	Bioactivity	Reference
Methanol	7	12.685	Hexadecanoic acid, methyl ester	C_17_H_34_O_2_	270.5	30.74	Antioxidative, Anti-inflammatory, Antimicrobial, Antifibrotic, Hepatoprotective	[26]
	9	13.808	9-Octadecenoic acid, methyl ester, (E)—(Elaidic acid, methyl ester)	C_19_H_36_O_2_	296.5	1.70	Antioxidative, Anti-inflammatory, Antimicrobial, Antiviral, Anticancerous, Antifungal, Antidiabetic, Antihypertensive	[27]
	15	18.850	1-Benzazirene-1-carboxylic acid, 2,2,5a-trimethyl-1a-[3-oxo-1-butenyl] perhydro-, methyl ester	C_15_H_23_NO_3_	265.35	0.67	Antitumor, Antioxidative, Antimicrobial, Antidiabetic, Anti-inflammatory	[28,29,30]
	16	19.045	1H-Indole, 1-methyl-2-phenyl-	C_15_H_13_N	207.27	2.14	Antioxidative, Antitumor	[31,32,33]
Ethanol	6	9.743	2,4-Di-tert-butylphenol	C_14_H_22_O	206.32	2.30	Antioxidative, Antibacterial, Antiviral, Anti-inflammatory, Cytotoxic and Antifungal	[34,35]
	7	10.423	Benzeneacetic acid, alpha.,3,4-tris[(trimethylsilyl)oxy]-, methyl ester	C_18_H_34_O_5_Si_3_	414.7	4.01	Anticancerous, Antimicrobial	[36]
	8	12.715	Hexadecanoic acid, methyl ester	C_17_H_34_O_2_	270.5	18.41	Antioxidative, Anti-inflammatory, Antimicrobial, Antifibrotic, Hepatoprotective	[26]
	10	13.887	Liguloxide	C_15_H_26_O	222.37	6.40	Antimicrobial, Anti-inflammatory, Antioxidative	[37,38]
	17	18.546	4(15)-Selinene-11,12-diol	C_15_H_26_O_2_	238.37	0.28	Antimicrobial, Anti-inflammatory, Antioxidative	[37,38]
	19	18.656	Benzo[h]quinoline, 2,4-dimethyl-	C_15_H_13_N	207.27	0.77	Antibacterial, Antiviral, Anti-inflammatory	[39]
	22	18.787	1H-Indole, 1-methyl-2-phenyl-	C_15_H_13_N	207.27	1.06	Antioxidative, Antitumor	[31,32,33]
	30	19.369	2-Methyl-7-phenylindole	C_15_H_13_N	207.27	1.91	Antioxidative, Antitumor	[31,32,33]
	34	19.516	1-Benzazirene-1-carboxylic acid, 2,2,5a-trimethyl-1a-[3-oxo-1-butenyl] perhydro-, methyl ester	C_15_H_23_NO_3_	265.35	1.00	Antitumor, Antioxidative, Antimicrobial, Antidiabetic, Anti-inflammatory	[28,29,30]

**Table 4:** List of identified bioactive compounds from LC-MS profiling of *Ulva lactuca.*

Extract	Peak	Proposed Compound	Class	Retention Time (min)	Observed Mass m/Z	Theoretical Mass m/Z	Molecular Formula	Bioactivity	Reference
Methanol	1	Kaempferol	Flavonoids	2.909	286.9	286.2363	C_15_H_10_O_6_	Antioxidative, Anti-inflammatory, Neuroprotective, Cardioprotective, Anticancerous	[40,41,42]
	2	Gallic acid	Benzene and substituted derivatives/Phenolic acid	3.856	170.9	170.12	C_7_H_6_O_5_	Anti-inflammatory, Antioxidative, Anticancerous, Antidiabetic, Cardioprotective, Antibacterial, Antifungal	[40,43]
	3	Caffeic acid sulphate	Cinnamic acid and derivatives/Phenolic acid	4.771	258.1	258.2	C_9_H_6_O_7S_	Antioxidative, Anti-inflammatory, Anticancerous	[43]
	4	Caffeic acid	Cinnamic acid and derivatives/Phenolic acid	7.206	180.1	180.1574	C_9_H_8_O_4_	Antioxidative, Anti-inflammatory, Anticancerous	[40,43]
	5	Quercetin	Flavonoids	7.493	302.2	302.2357	C_15_H_10_O_7_	Antioxidative, Anti-inflammatory, Anticancerous, Cardioprotective	[41,44,45]
	6	4-hydroxybenzoic acid	Benzene and substituted derivatives/Phenolic acid	7.790	138.0	138.122	C_7_H_6_O_3_	Antioxidative, Antibacterial, Antifungal, Anticancerous	[40,43]
	8	Syringic acid	Benzene and substituted derivatives/Phenolic acid	8.815	198.1	198.1740	C_9_H_10_O_5_	Antioxidative, Anti-inflammatory, Hepatoprotective, Anticancerous, Antidiabetic, Cardioprotective, Antimicrobial, Neuroprotective	[40,43]
	9	Quercitrin	Flavonoids	9.091	448.2	448.3769	C_21_H_20_O_11_	Anti-inflammatory, Antioxidative, Antiallergic, Neuroprotective	[46]
	10	Vanillin	Phenols	9.796	152.9	152.1473	C_8_H_8_O_3_	Anti-inflammatory, Antioxidative, Anticancerous, Antibacterial, Neuroprotective	[43]
Ethanol	5	Quercetin	Flavonoids	7.570	302.1	302.2357	C_15_H_10_O_7_	Antioxidative, Anti-inflammatory, Anticancerous, Cardioprotective	[41,44,45]
	7	4-hydroxybenzoic acid	Benzene and substituted derivatives/Phenolic acid	7.912	138.0	138.122	C_7_H_6_O_3_	Antioxidative, Antibacterial, Antifungal, Anticancerous	[40,43]
	8	Vanillic acid	Benzene and substituted derivatives/Phenolic acid	8.022	168.1	168.1480	C_8_H_8_O_4_	Antioxidative, Anti-inflammatory, Antimicrobial, Antidiabetic, Neuroprotective, Anticancerous	[40]
	10	Caffeic acid	Cinnamic acid and derivatives/Phenolic acid	8.341	180.1	180.1574	C_9_H_8_O_4_	Antioxidative, Anti-inflammatory, Anticancerous	[40,43]
	11	Gallic acid	Benzene and substituted derivatives/Phenolic acid	8.518	170.9	170.12	C_7_H_6_O_5_	Anti-inflammatory, Antioxidative, Anticancerous, Antidiabetic, Cardioprotective, Antibacterial, Antifungal	[40,43]
	12	Syringic acid	Benzene and substituted derivatives/Phenolic acid	8.826	198.1	198.1740	C_9_H_10_O_5_	Antioxidative, Anti-inflammatory, Hepatoprotective, Anticancerous, Antidiabetic, Cardioprotective, Antimicrobial, Neuroprotective	[40,43]
	13	Quercitrin	Flavonoids	9.091	448.2	448.3769	C_21_H_20_O_11_	Anti-inflammatory, Antioxidative, Antiallergic, Neuroprotective	[46]
Aqueous	4	Kaempferol	Flavonoids	2.777	286.9	286.2363	C_15_H_10_O_6_	Antioxidative, Anti-inflammatory, Neuroprotective, Cardioprotective, Anticancerous	[40,41,42]
	5	Quercetin	Flavonoids	3.361	302.2	302.2357	C_15_H_10_O_7_	Antioxidative, Anti-inflammatory, Anticancerous, Cardioprotective	[41,44,45]
	6	Syringic acid	Benzene and substituted derivatives/Phenolic acid	3.790	198.0	198.1740	C_9_H_10_O_5_	Antioxidative, Anti-inflammatory, Hepatoprotective, Anticancerous, Antidiabetic, Cardioprotective, Antimicrobial, Neuroprotective	[40,43]
	8	Vanillic acid	Benzene and substituted derivatives/Phenolic acid	5.146	168.1	168.1480	C_8_H_8_O_4_	Antioxidative, Anti-inflammatory, Antimicrobial, Antidiabetic, Neuroprotective, Anticancerous	[40]
	10	4-hydroxybenzoic acid	Benzene and substituted derivatives/Phenolic acid	7.328	138.0	138.122	C_7_H_6_O_3_	Antioxidative, Antibacterial, Antifungal, Anticancerous	[40,43]
	13	Caffeic acid	Cinnamic acid and derivatives/Phenolic acid	8.165	180.1	180.1574	C_9_H_8_O_4_	Antioxidative, Anti-inflammatory, Anticancerous	[40,43]
	14	Gallic acid	Benzene and substituted derivatives/Phenolic acid	8.518	170.0	170.12	C_7_H_6_O_5_	Anti-inflammatory, Antioxidative, Anticancerous, Antidiabetic, Cardioprotective, Antibacterial, Antifungal	[40,43]
	15	Quercitrin	Flavonoids	9.234	448.2	448.3769	C_21_H_20_O_11_	Anti-inflammatory, Antioxidative, Antiallergic, Neuroprotective	[46]

**Table 5:** Phytochemical and antioxidant profile of *Ulva lactuca.*

Sr. No.	Solvent	TPC (mg GAE/100 mL)	TFC (µg QE/mL)	DPPH (%)	FRAP (µmol/g)
1	Methanol	1965.3 ± 0.7 ^b^	380.5 ± 0.8 ^b^	71.5 ± 0.02 ^b^	110.5 ± 0.1 ^b^
2	Ethanol	2014.6 ± 0.9 ^a^	750.5 ± 0.7 ^a^	77.7 ± 0.02 ^a^	95.8 ± 0.1 ^c^
3	Aqueous	2015.1 ± 0.6 ^a^	145.1 ± 0.8 ^c^	66.5 ± 0.10 ^c^	170.1 ± 0.1 ^a^
*p*-value		0.000 *	0.000 *	0.000 *	0.000 *

TPC: total phenolic content; TFC: total flavonoid content; DPPH: 2,2-diphenyl-1-picrylhydrazyl; FRAP: ferric reducing antioxidant power. All values are expressed as mean ± standard deviation (n = 3). Various superscript letters within the same column indicate statistically significant differences between the groups. * Statistically significant *p*-value ≤ 0.05.

**Table 6:** Impact of various interventions on body weight.

Groups	Week 1	Week 2	Week 3	Week 4	*p*-Value
I	172.5 ± 9.4 ^d^	188.3 ± 10.3 ^c^	205.8 ± 11.1 ^b^	225.0 ± 13.0 ^a^	0.000 *
II	184.2 ± 11.6 ^a^	173.3 ± 10.8 ^a^	171.7 ± 11.7 ^a^	181.7 ± 12.1 ^a^	0.201
III	191.7 ± 5.3 ^b^	176.2 ± 5.5 ^c^	193.8 ± 5.8 ^b^	221.3 ± 5.4 ^a^	0.000 *
IV	185.8 ± 7.6 ^a^	164.5 ± 9.4 ^b^	168.7 ± 8.2 ^b^	172.3 ± 8.8 ^b^	0.002 *

I: Normal Control; II: Disease Control; III: Standard Drug; IV: *Ulva lactuca*. All the data are represented as mean ± standard deviation in grams of body weight. Multiple superscripts present in the same row signify statistically significant variation in mean weight of the rats throughout the trial. * Statistically significant *p*-value ≤ 0.05.

**Table 7:** Impact of various interventions on serum lipid profile.

Groups	Total Cholesterol (mg/dL)	TGs (mg/dL)	HDL (mg/dL)	LDL (mg/dL)	VLDL (mg/dL)	Chol/HDL Ratio
I	87.4 ± 6.6 ^d^	96.1 ± 7.2 ^c^	29.2 ± 5.1 ^a^	39.0 ± 9.3 ^d^	19.2 ± 1.6 ^c^	3.1 ± 0.7 ^c^
II	209.9 ± 8.4 ^a^	136.4 ± 8.7 ^a^	14.1 ± 2.8 ^b^	168.6 ± 7.5 ^a^	27.3 ± 1.7 ^a^	15.3 ± 2.8 ^a^
III	124.5 ± 6.2 ^c^	106.4 ± 7.8 ^b^	27.5 ± 7.5 ^a^	75.7 ± 10.9 ^c^	21.3 ± 1.6 ^b^	4.8 ± 1.3 ^b,c^
IV	133.2 ± 5.7 ^b^	99.2 ± 7.7 ^b,c^	22.4 ± 6.5 ^a^	90.9 ± 7.9 ^b^	19.8 ± 1.5 ^b,c^	6.4 ± 1.9 ^b^
*p*-value	0.000 *	0.000 *	0.001 *	0.000 *	0.000 *	0.000 *

I: Normal Control; II: Disease Control; III: Standard Drug; IV: *Ulva lactuca*. TGs: triglycerides; HDL: high-density lipoprotein; LDL: low-density lipoprotein; VLDL: very-low-density lipoprotein. All the data are represented as mean ± standard deviation. Multiple superscripts present in the same column signify statistically significant disparities among the groups. * Statistically significant *p*-value ≤ 0.05.

**Table 8:** Impact of various interventions on atherogenic indexes.

Groups	Atherogenic Index of Plasma	Castelli’s Risk Index I	Castelli’s Risk Index II	Atherogenic Coefficient
I	0.52 ± 0.08 ^c^	3.08 ± 0.65 ^c^	1.41 ± 0.54 ^c^	2.08 ± 0.65 ^c^
II	0.99 ± 0.09 ^a^	15.34 ± 2.83 ^a^	12.34 ± 2.42 ^a^	14.34 ± 2.82 ^a^
III	0.60 ± 0.11 ^b,c^	4.79 ± 1.29 ^b,c^	2.98 ± 1.12 ^b,c^	3.79 ± 1.29 ^b,c^
IV	0.66 ± 0.14 ^b^	6.38 ± 1.92 ^b^	4.42 ± 1.59 ^b^	5.38 ± 1.92 ^b^
*p*-value	0.000 *	0.000 *	0.000 *	0.000 *

I: Normal Control; II: Disease Control; III: Standard Drug; IV: *Ulva lactuca*. All the data are represented as mean ± standard deviation. Various superscripts present in the same column signify statistically significant disparities among the groups. * Statistically significant *p*-value ≤ 0.05.

**Table 9:** Impact of various interventions on hepatic function panel.

Groups	Total Bilirubin (mg/dL)	ALT (U/L)	AST (U/L)	ALP (U/L)	GGT (U/L)	Total Protein (g/dL)	Albumin (g/dL)	Globulin (g/dL)	A/G Ratio
I	0.43 ± 0.09 ^c^	28.47 ± 2.63 ^d^	84.63 ± 2.74 ^d^	232.50 ± 8.18 ^d^	3.04 ± 0.36 ^c^	7.09 ± 0.19 ^a^	3.59 ± 0.43 ^a^	3.51 ± 0.52 ^a^	1.07 ± 0.33 ^a^
II	1.92 ± 0.49 ^a^	158.50 ± 2.26 ^a^	310.52 ± 5.45 ^a^	416.92 ± 4.87 ^a^	5.99 ± 0.49 ^a^	5.25 ± 0.10 ^b^	2.44 ± 0.20 ^c^	2.81 ± 0.17 ^b^	0.88 ± 0.12 ^a,b^
III	1.01 ± 0.13 ^b^	51.32 ± 3.28 ^c^	90.77 ± 4.39 ^c^	259.85 ± 7.89 ^c^	3.64 ± 0.34 ^b^	6.92 ± 0.47 ^a^	3.02 ± 0.33 ^b^	3.89 ± 0.49 ^a^	0.79 ± 0.15 ^b^
IV	0.96 ± 0.11 ^b^	62.57 ± 6.04 ^b^	117.87 ± 5.97 ^b^	333.17 ± 4.02 ^b^	3.76 ± 0.32 ^b^	6.88 ± 0.38 ^a^	3.09 ± 0.29 ^b^	3.79 ± 0.27 ^a^	0.82 ± 0.09 ^ab^
*p*-value	0.000 *	0.000 *	0.000 *	0.000 *	0.000 *	0.000 *	0.000 *	0.000 *	0.083

I: Normal Control; II: Disease Control; III: Standard Drug; IV: *Ulva lactuca*; ALT: alanine aminotransferase; AST: aspartate aminotransferase; ALP: alkaline phosphatase; GGT: gamma-glutamyl transferase; A/G ratio: albumin/globulin ratio. All the data are represented as mean ± standard deviation. Various superscripts present in the same column signify statistically significant disparities among the groups. * Statistically significant *p*-value ≤ 0.05.

## Data Availability

The original contributions presented in this study are included in the article/supplementary material. Further inquiries can be directed to the corresponding author.

## Supplementary Materials

The following supporting information can be downloaded at: https://www.mdpi.com/article/10.3390/foods15152695/s1, Table S1. Proximate Analysis Parameters and Corresponding Formulas; Table S2. Formulas Used for the Calculation of Atherogenic Indices; Figure S1. Gas-Chromatography-Mass Spectrometry (GC-MS) Profile of Methanolic (A) and Ethanolic Extract (B) of Ulva lactuca; Figure S2. Liquid-Chromatography-Mass Spectrometry (LC-MS) Profile of Methanolic Extract of Ulva lactuca, Peak 1: Kaempferol, Peak 2: Gallic acid, Peak 3: Caffeic acid sulphate, Peak 4: Caffeic acid, Peak 5: Quercetin, Peak 6: 4-hydroxybenzoic acid, Peak 8: Syringic acid, Peak 9: Quercitrin, Peak 10: Vanillin; Figure S3. Liquid-Chromatography-Mass Spectrometry (LC-MS) Profile of Ethanolic Extract of Ulva lactuca, Peak 5: Quercetin, Peak 7: 4-hydroxybenzoic acid, Peak 8: Vanillic acid, Peak 10: Caffeic acid, Peak 11: Gallic acid, Peak 12: Syringic acid, Peak 13: Quercitrin; Figure S4. Liquid-Chromatography-Mass Spectrometry (LC-MS) Profile of Aqueous Extract of Ulva lactuca, Peak 4: Kaempferol, Peak 5: Quercetin, Peak 6: Syringic acid, Peak 8: Vanillic acid, Peak 10: 4-hydroxybenzoic acid, Peak 13: Caffeic acid, Peak 14: Gallic acid, Peak 15: Quercitrin.
