# Supplementary material for: Evaluation of the Antioxidative and Hepatoprotective Activity of Ulva lactuca in a CCl4-Induced Liver Injury Model
Source: Foods. 2026 Jul 30;15(15):2695. doi: 10.3390/foods15152695 (PMC13464905; doi:10.3390/foods15152695)
Supplement: Supplementary file 1 [file foods-15-02695-s001.zip › foods-4274337-supplementary.pdf]

Table S1 Proximate Analysis Parameters and Corresponding Formulas

Proximate Components (%)	Formula
Moisture	$= \frac{wt.of\ fresh\ sample - wt.of\ dried\ sample}{wt.of\ fresh\ sample} \times 100$
Total Ash	$= \frac{(wt.\ of\ ash + Crucible) - wt.\ of\ crucible}{wt.\ of\ sample} \times 100$
Crude Protein	***Nitrogen (%)*** $= \frac{Vol.Of\ 0.1\ N\ H_2SO_4 \times Vol.of\ dilution \times 0.0014}{Wt.\ of\ sample \times Vol.\ of\ dillution} \times 100$<br>***Crude Protein (%) =*** *Nitrogen (%) × 6.25*
Crude Fat	$= \frac{weight\ of\ timple\ before\ drying - weight\ of\ timble\ after\ drying}{weight\ of\ sample} \times 100$
Crude Fiber	$= \frac{wt.of\ crucible\ with\ sample\ before\ ashing - wt.of\ crucible\ with\ ash\ after\ ashing}{wt.of\ sample} \times 100$
NFE or Nitrogen Free Extract	$= 100 - (Total\ Moisture\ \% + Total\ Ash\ \% + Crude\ Protein\ \% + Crude\ Fat\ \% + Crude\ Fiber\ \%)$

Table S2 Formulas Used for the Calculation of Atherogenic Indices

Components	Formula
**Atherogenic Index of Plasma (AIP)**	$\log 10 \left(\frac{TGs}{HDL}\right)$
**Castelli's Risk Index I (CRI-I)**	$\frac{Total\ Cholesterol}{HDL}$
**Castelli's Risk Index II (CRI-II)**	$\frac{LDL}{HDL}$
**Atherogenic Coefficient (AC)**	$\frac{Total\ Cholesterol - HDL}{HDL}$

TGs: *Triglycerides;* ***HDL:*** *High-Density Lipoprotein;* ***LDL:*** *Low-Density Lipoprotein*
